# Using Immersive Virtual Reality in an Online Biology Course

**DOI:** 10.1007/s41979-023-00095-9

**Published:** 2023-05-12

**Authors:** Ania A. Majewska, Ethell Vereen

**Affiliations:** 1 Department of Physiology and Pharmacology, College of Veterinary Medicine, University of Georgia, GA 30602 Athens, USA; 2 Department of Biology, Morehouse College, Atlanta, GA 30314 USA

**Keywords:** VR, HBCU, Remote learning, Oculus headset, Undergraduate, Non-major

## Abstract

Interest in virtual reality (VR) for teaching and learning in higher education is growing, given its many potential applications. VR offers a socially interactive environment with novel ways to engage students with materials, objects, and activities and provide students with experiences such as “field trips” that would be otherwise very difficult. Preliminary work indicates overall positive gains in student learning across disciplines compared to other technology and traditional techniques, although more studies are needed to better our understanding of this tool. We employed an “immersive” VR (with a head-mounted display) in an online course which provided students with the opportunity to interact with peers and engage in activities. We asked about perceptions of the learning experience with the technology and how using VR impacts students’ performance. We also noted the benefits and challenges of VR in an online course. Students perceived VR as a helpful component of the course, although performance on the cardiovascular unit assessment did not differ compared to the previous semester without VR.

**Supplementary Information:**

The online version contains supplementary material available at 10.1007/s41979-023-00095-9.

## Introduction

Virtual reality (VR) technology is increasingly being considered by educators to be a pedagogical tool that can be adopted for improved student learning (Fowler, 2015; Kwun et al., 2019; Matovu et al., 2022). Although it is still primarily considered for entertainment (e.g., gaming), VR has long been recognized as a teaching tool in the military for flight simulations and scenario exercises (Hawkins, 1995). VR can provide an interactive and engaging experience for students via a desktop computer, tablet, or smartphone, or the use of a head-mounted display, such as Meta’s (formerly Facebook) Oculus Quest 2 (https://www.oculus.com) (Wohlgenannt et al., 2020). With the head-mounted display, students can only see the virtual environment with a 360° view and sound, which gives the users a strong sense of presence and is referred to as “immersive” VR (Wohlgenannt et al., 2020). Furthermore, headset-delivered VR allows students to interact with peers, manipulate and build new objects, and virtually “touch” objects that they might not have access to in their classroom due to various limiting factors. For instance, students can travel through parts of the human body or visit sites across the globe. Without VR, providing similar learning experiences to students would be challenging in real life (Dalgarno & Lee, 2010).

In higher education, VR has been most widely employed in engineering courses to enhance procedural-practical knowledge (i.e., knowing how to perform a task) (Radianti et al., 2020), as well as in medical fields for teaching anatomy and skill training (e.g., surgical performance) (Portelli et al., 2020). Yet, examination of the effectiveness of VR (via desktop and headset) in enhancing student outcomes in higher education shows mixed results (Coban et al., 2022; Matovu et al., 2022; Wu et al., 2020). A meta-analysis examining studies focused on medical student gains in anatomy knowledge with VR showed a moderate improvement in test scores compared to students who used textbooks, 2D images, or 3D models (Zhao et al., 2020). On the other hand, meta-analyses of student learning across various disciplines showed no difference in the use of VR versus traditional training methods (Kaplan et al., 2021). It is important to note that the number of studies remains low, and conclusions about the effectiveness of the technology are premature. Furthermore, the study design and learning goals vary considerably from gaining a procedural skill to content knowledge (Coban et al., 2022), making general conclusions about VR in higher education difficult.

Interestingly, the use of VR in education has shown the greatest learning gains in the architecture, engineering, and geometry fields, where the use and practice of spatial abilities are important (Coban et al., 2022). Thus, VR might be particularly beneficial for learning about 3D objects that are oversimplified in textbooks and graphical depictions used for teaching (e.g., the human heart). Many of the misconceptions that persist through professional studies about the cardiovascular system might stem from oversimplification of 2D visual aids (Ahopelto et al., 2011; Kaufman et al., 2013; Södervik et al., 2019). Indeed, spatial relationships of anatomical structures in complex 3D objects, such as the heart, are challenging to learn (Nakai et al., 2022). Given that VR provides 3D visual displays which can be manipulated and observed from various angles, using this technology for teaching and learning anatomy and physiology might hold advantages compared to traditional 2D interfaces (Maresky et al., 2019; Shelton & Hedley, 2004). In addition, using VR with 3D representations might assist in the development of spatial thinking (Carbonell Carrera & Bermejo Asensio, 2017), which is considered a critical skill for students in STEM (Uttal et al., 2013). Considering these benefits, exploring the potential benefits of immersive VR in learning about complex structures is imperative.

Effective integration of a new technology in a course necessitates student buy-in. For example, when computers and the Web first made their way to the classroom, student attitudes towards the technology in respect to learning predicted their adoptions (Halpin & Myers, 2002; Huang & Liaw, 2005). In relation to immersive VR as a novel educational tool, previous studies indicate that positive attitudes and perceptions towards it (Domingo & Bradley, 2018; Kavanagh et al., 2017) can in turn positively impact student gains and performance (Georgiou et al., 2021; Tsivitanidou et al., 2021). Yet, using new tools which are highly demanding for students to learn to use and navigate, as may occur with using headsets with controls for immersive VR, is likely to be met with tepid enthusiasm. Given that immersive VR is known to cause cybersickness, or nausea and dizziness, for some users (Nesbitt et al., 2017), student perceptions of the technology are likely mixed. Despite the negative sensations learners can experience, recent work suggests that students perceive immersive VR in online courses positively (Duncan‐Vaidya & Stevenson, 2021). Thus, assessing student perceptions of immersive VR when integrated into learning experiences, particularly in novel environments such as remote courses, warrants assessment and further exploration.

A new and considerably less common application of VR is in online courses (Atkins et al., 2020). With the increased use of the online environment for teaching since the onset of the COVID-19 pandemic, yet dwindling enthusiasm for the use of video conferencing, “Zoom fatigue” (Nadler, 2020), and challenges associated with distractions and focus (Peper et al., 2021), solutions for engaging students online are needed. Given the possibility of immersive VR to provide a socially interactive environment, the technology might therefore offer a solution for increasing student engagement in online courses (Fromm et al., 2021) and provide much-needed social interactions in a remote setting. Furthermore, immersive VR is expected to have positive impact on student learning of complex 3D structures, yet such effects remain largely unexplored in online classrooms.

The research questions (RQs) guiding this work are:

- RQ1: What are student perceptions of immersive VR in an introductory non-major biology course delivered *online*?
- RQ2: Is there evidence that VR positively impacts student performance in an introductory non-major biology course delivered *online*?

We gauged student perceptions of VR technology using short surveys consisting of closed-ended, Likert scale responses. We examined whether a lesson in VR on the cardiovascular system resulted in better student performance on an assessment compared to the previous semester which was delivered online but did not include VR sessions. Finally, we identified unique ways and opportunities in which VR can be utilized, as well as the drawbacks and challenges of an online biology course.

## Methods

### Participants

We incorporated VR activities in our first-year non-major biology course with 20 undergraduate students, most of whom were first-year and transfer students. All students were males enrolled in Men’s Health (Majewska et al., 2022), a course at Morehouse College, Atlanta, GA, USA, a historically black college.

### Procedures

We launched the immersive VR activities in Spring 2021. As with all other course activities, we used a backward course design to develop the lesson plans based on student learning outcomes for the VR sessions (see examples in supplementary information). The course was taught online due to restrictions associated with COVID-19. Students registered for the courses without the prior knowledge that the course would include activities facilitated in VR and none had used VR headsets for educational purposes prior to the course. The Institutional Review Board approval (IRB—570,002,057) was obtained from Morehouse College for this study.

**Fig. 1:**
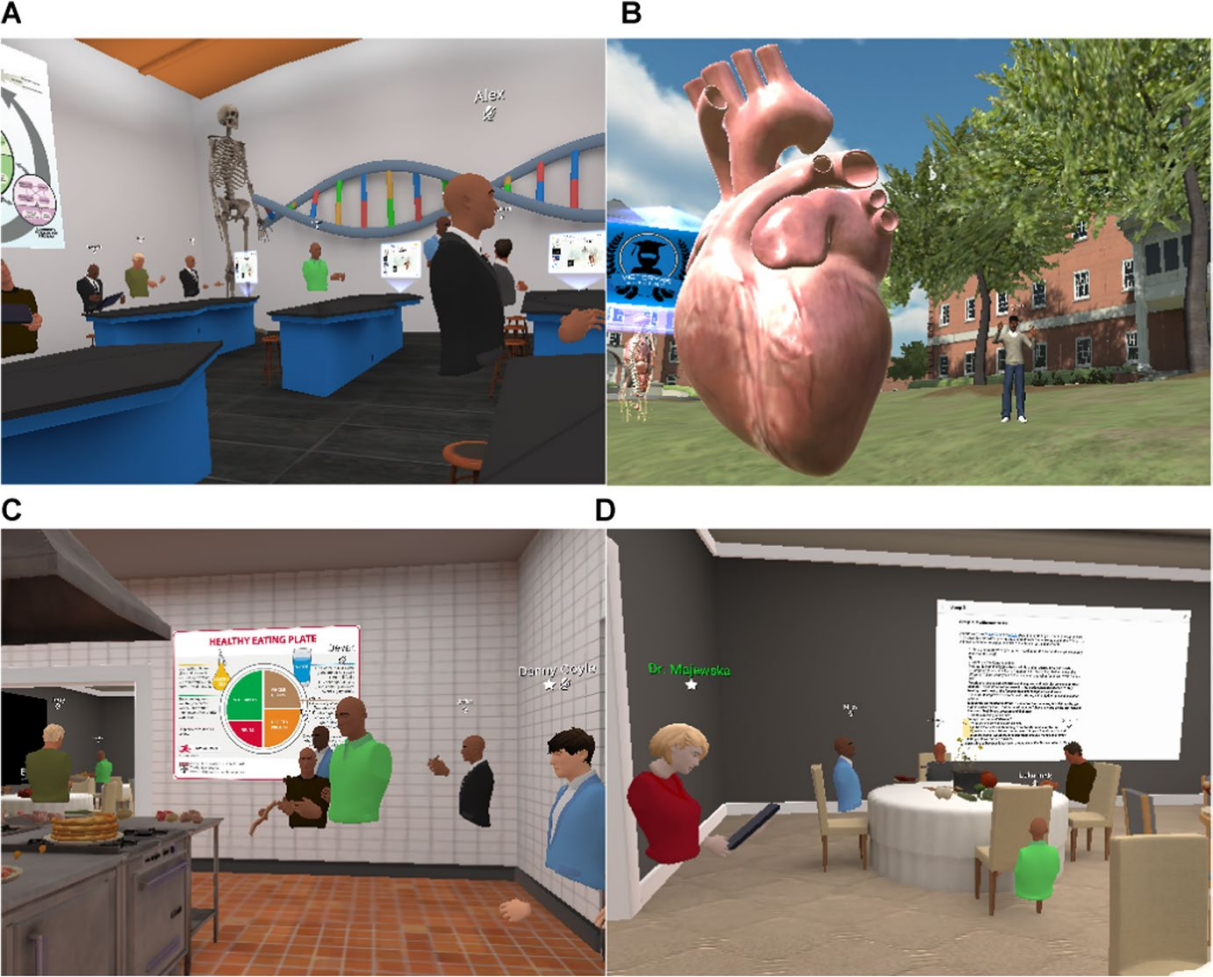
Examples of VR environments and objects. **A** Students seated in a “wet lab” classroom. **B** Examining the human heart on the virtual Morehouse campus quad. **C–D** Healthy diets lab activity in the industrial kitchen and adjacent dining room. Note that the avatars consist of torsos and hands; only the main host has an avatar with a full body (image **B**)

Students and instructors used the Oculus Quest 2 headset and associated handheld controllers, powered by the Qualcomm Snapdragon XR2 Platform. The headsets were shipped directly to each student. The objects, digital Morehouse campus, and other VR environments (e.g., wet lab; Fig. 1A) used in the course were created by content provider VictoryXR Inc (https://www.victoryxr.com/), hosted on the ENGAGE platform (https://engagevr.io/). VictoryXR staff facilitated training sessions with the instructors both as a group and independently to focus on specific aspects of each activity. Training sessions were necessary for instructors to develop facilitator skills via the handheld controllers and classroom management tools (teleporting, summoning, 3D audio). Additional online training by VictoryXR was available on YouTube for independent learning. We launched a “soft” start of the VR environments with VictoryXR staff prior to instruction with students, which allowed us to gain additional comfort with VR classroom management and object manipulation. VictoryXR assisted with the onboarding of students and “sat in” on initial sessions to offer students additional support and provide technical assistance.

We announced the upcoming VR sessions during class meetings on the online learning management system (Blackboard), and via email ahead of class time. Most of the VR sessions took place during the 2-h lab section of the course, which provided us sufficient time for any potential issues that could arise and for additional time to increase VR stamina given that initial use of VR can result in dizziness and motion sickness (Park & Lee, 2020). For each VR session, we first met on Zoom, provided an overview of VR activities, and transitioned to the headsets. We referenced the presence in VR environments as being on the digital Morehouse campus. The platform we used required an instructor-created link to join the VR session. For the first VR session, students were joined by VictoryXR staff on the VR Morehouse campus quad (a VR environment) and were given instructions and practice time on how to use the handheld controllers to move around the VR environment and add, resize, and manage VR objects.

We used VR to deliver several activities, including lessons on HeLa cells, cancer, the cardiovascular system, healthy foods and nutrition, the scientific method, and group project presentations (Fig. 1; for example lesson plans; see supplementary information). Each session was between 15 and 30 min and started with the students in a VR environment of the instructor’s choice (e.g., industrial kitchen, Fig. 1). Besides utilizing VR environments, we incorporated a “field trip” to the ocean in which students were asked to identify dependent and independent variables of a MythBusters (television program) experiment. This activity was part of our overarching aim for students to understand the scientific method. As part of the activity, students were able to virtually experience swimming with sharks in the “MythBusters: Underwater Shark Experiment. Are sharks repelled by the essence of dead sharks?” which can be viewed in the headset as a 360° video (https://www.youtube.com/watch?v=g_WZncx-Baw). The video was embedded into the VR environment, and viewing was controlled by the instructor, such that the video could be paused and the instructor verbally asked the students questions. Following the class time with a VR session, instructors reflected on the activity and recorded notes on the benefits and challenges encountered.

For the cardiovascular system unit of the course, we held a session in VR examining the anatomy of the heart. We began the session with students on the VR campus lawn where an enlarged transparent human body with a visible pulmonary and cardiovascular system was displayed. We also included a standalone enlarged human heart (Fig. 1B). We first provided a brief verbal description of the function of the cardiovascular system and explained the flow of oxygenated and deoxygenated blood through the system using the transparent human body as a visual aid. Next, we invited students to “walk” inside the enlarged heart while we pointed out the main anatomical features (e.g., atriums, ventricles, valves) and explained blood flow through the heart. Students were then asked to explore the exterior and interior heart anatomy.

### Data Collection and Analysis

#### RQ1: What Are Student Perceptions of VR in an Introductory Non-major Biology Course Delivered Online?

We gauged student perceptions of VR in this course with short in-class surveys and an end-of-semester evaluation consisting of closed-ended Likert scale responses (Table 1). Participation in the surveys was voluntary. For in-class surveys, in the last 5–10 min, students were asked to answer questions (administered in VR) to gauge student perceptions of three sessions (weeks 4 to 6; Table 1). Survey questions asked during VR sessions were not validated. We did not administer surveys during each VR session in order to not overwhelm the students. To assess whether survey scores for each question differed between the three weeks, we used a simple linear regression with the score as a response and the week of the session as an explanatory variable.

**Table 1:** Closed-ended survey questions and evaluation prompts administered to students to gauge their perceptions of VR experiences

a. Questions (on weeks 4–6)	Likert scale
Q1: How smooth was the process to get onto the digital Morehouse campus?	1 to 4: 1 hardest to 4 easiest
Q2: How much problem did you have with nausea while in the headset?	1 to 4: 1 no problem to 4 very bad
Q3: How was the use of virtual reality in conveying the concepts of the class?	1 to 4: 1 not-useful to 4 very-useful
b. Questions (on last VR session)	Likert scale
How was the use of virtual reality in conveying the concepts of HeLa cells (guest lecturer) (S1)?	1 to 4: 1 not-useful to 4 very-useful
How was the use of virtual reality in conveying the concepts of cancer (S2)?	
How was the use of virtual reality in conveying the concepts of cardiovascular system (heart lab) (S3)?	
How was the use of virtual reality in conveying the concepts of healthy food and nutrition (diet lab) (S4)?	
How was the use of virtual reality in conveying the concepts of scientific method (scientific method lab) (S5)?	
How would you rate your overall experience with VR in this course?	1 to 4: 1 terrible to 4 awesome
c. Prompts (end-of-semester evaluations)	Likert scale
Rate the effectiveness of using VR environments in facilitating your learning	not at all effective, slightly effective, moderately effective, very effective, extremely effective, and not applicable
Rate the effectiveness of using VR field trips in facilitating your learning	
Rate the effectiveness of using VR to create and manipulate objects in facilitating your learning	
Rate the effectiveness of using VR for group activities and presentations in facilitating your learning	
Rate the effectiveness of using VR, the overall experience, in facilitating your learning	

During our last VR session, we administered an additional six-question survey to ask students about their perceptions of the previous VR sessions (Table 1). We also used the end-of-semester course evaluations which were administered to in part assess student perception of the overall VR experience (Table 1). The course evaluation questionnaire is based on the IDEA Student Ratings of Instruction and is validated (Benton & Li, 2017). The evaluations were distributed via Blackboard prior to the last class session.

#### RQ2: Is There Evidence that VR Positively Impacts Student Performance in an Introductory Non-major Biology Course Delivered Online?

To assess student learning of the cardiovascular system, 3 days following the cardiovascular lesson in VR, students completed a quiz (eight questions administered on Blackboard). The same quiz was completed by students in the previous semester, who received a lecture on the cardiovascular system delivered via PowerPoint presentation on Zoom. We compared two semester scores students earned using a Wilcoxon test since the scores were not normally distributed as revealed by the Shapiro–Wilk normality test (semester 1: *W* = 0.84, *p* = 0.006, semester 2: *W* = 0.73, *p* < 0.001*)*. All statistical analyses were performed using R programming (R Core Team, 2022).

## Results

### RQ1: What Are Student Perceptions of VR in an Introductory Non-major Biology Course Delivered Online?

**Fig. 2:**
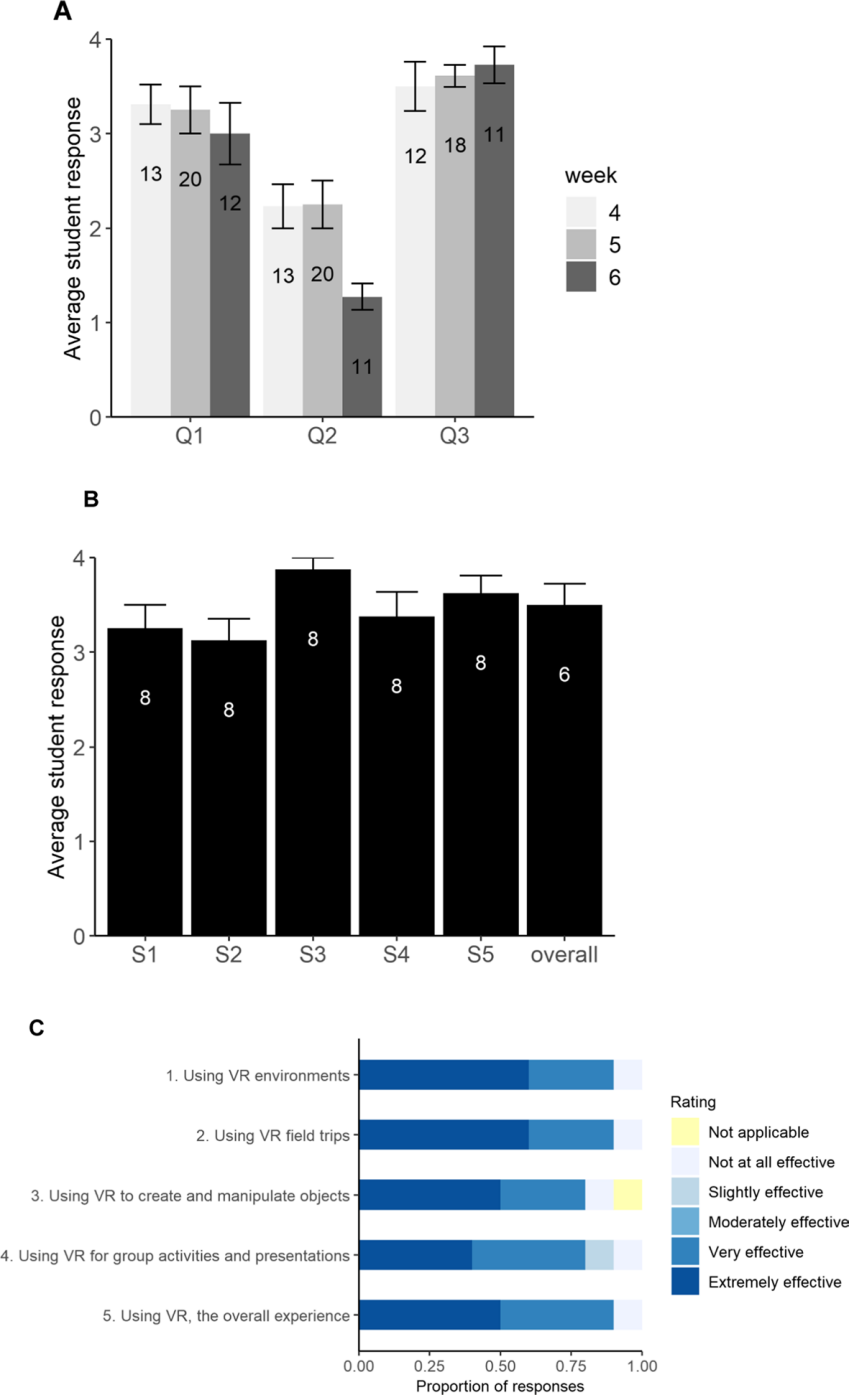
Average student responses to survey questions administered during virtual reality sessions on **A** weeks 4 to 6 and on the **B** last virtual reality session of the semester. Numbers inside the bar represent the number of student respondents. Error bars represent ± standard error. **C** Results of end-of-semester evaluations (administered on Blackboard) on the perceptions of the immersive virtual reality class experience. Survey and evaluation questions are provided in Table 1. Ten students completed the end-of-semester course evaluations

The in-class surveys which we administered at the end of a VR session in the headsets received eleven to twenty student responses. Results suggest that students did not find the transition to headsets to be difficult (Q1; Fig. 2A). Also, students scored nausea as a moderate problem (Q2), with scores significantly decreasing by week 6 (*t* =  − 2.56, df = 41, *p* = 0.01; Fig. 2A). Finally, student indicated VR was useful in conveying class concepts for the given week (Q3; Fig. 2A). Average scores did not differ between sessions for Q1 and Q3 (*p* > 0.05). The in-class survey administered during the last VR session received six to eight student responses and showed that the respondents found VR generally useful for the course (Fig. 2B).

Ten of the twenty students enrolled in the course completed end-of-semester evaluations. Results suggested that respondents found the different components of the VR session to be effective in their learning (Fig. 2C). Overall, respondents indicated VR was a helpful component of the course.

### RQ2: Is There Evidence That VR Positively Impacts Student Performance in an Introductory Non-major Biology Course Delivered Online?

The average score on a cardiovascular assessment for students in a course with VR was 8.50 points (*n* = 14 students; SD = 1.61) and without VR was 7.44 points (*n* = 18 students; SD = 2.18) out of 10 maximum points. The difference in average points earned was not statistically significant (*W* = 95, *p* = 0.22).

### Instructors’ Notes on Benefits and Challenges

We encountered various challenges as well as unique opportunities in which VR can be applied in an online biology course (for summary see Table 2). We identified several features of immersive VR as improvements to our instruction in a remote environment. Specifically, VR facilitated novel and unique learning experiences, such as a “field trip” to the ocean to observe a shark experiment and explore the inside of a human heart, which are not readily feasible in a traditional or online course. In addition to VR’s benefit of experiential learning in an online course, compared to using a video conferencing platform (e.g., Zoom), we noted that social interactions and collaborative work were more “realistic” in immersive VR. For instance, VR did not require the separation of students into break-out rooms for group work.

**Table 2:** Benefits and opportunities as well as challenges and limitations we identified with delivering immersive VR activities online

*Benefits and opportunities*	*Challenges and limitations*
• Student enthusiasm and curiosity to use new technology in a course	• Monetary cost associated with headsets and shipments to students
• More authentic interactions while online (e.g., all participants can speak at the same time but only those who are ‘close’ are audible)	• Requirement of reliable internet and minimum speeds of about 25Mbit/s
• Designing novel classroom environments and objects (e.g., a wet lab with various anatomical structures)	• Time-consuming onboarding to learn how to use the headset, controllers, and the hosting platform as well as to build and manipulate environments and objects
• Freedom to explore and manipulate objects by scaling, resizing, and walking through the objects	• *Some physical discomfort in the form of dizziness as avatar moves
• Visiting VR environments (pre-existing or built by other instructors) that represent other parts of the world (e.g., ancient Egyptian tomb)	• Creating VR environments and novel or complex objects may require a developer
• Viewing 360 videos (e.g., shark experiment) as field trips	• *Multiple steps are needed for student transition from Zoom to headsets: registration online using a computer, login to the platform, and selection of the correct session
• Sessions can be recorded, posted, and viewed later while retaining the ability to move around the space, interact with the environment and other viewers	• *Limited number of participants that can be recorded in a session
• Students can be quickly summoned, restricted to their seating area, and muted	• *Limited note-taking. For typing, handheld controllers are used for clicking and selecting one letter at a time

star symbol (*) indicates a matter that we encountered with the platform we used

We also noted the challenges of using VR in an online course. Learning how to use the technology, navigate the VR environment, and manipulate objects was time-consuming. Time was also lost during transitions into the VR headsets due to multiple steps required for registration and logins to the app; however, we note that this challenge might be specific to the platform we used. Also, internet availability, reliability, and sufficient speed (about 25Mbit/s) were all essential for participation in VR activities. Thus, any problems with the internet caused disturbances to VR sessions for students.

## Discussion

### RQ1: Student Perceptions of VR

We implemented a pilot study with immersive VR activities in an online biology non-major course for undergraduate students to ask students about their perceptions of the new technology. We found that students rated it as a useful and effective tool in their learning, despite some students experiencing the negative effects of cybersickness. We note that the survey questions asked during VR sessions were not validated and should be interpreted cautiously. Yet, given the positive responses noted in evaluations, which were validated, it suggests the students conveyed overall positive perceptions of VR activities in the online course. In agreement, previous work indicates students find VR engaging and express positive attitudes and perceptions towards the use of technology in a course (Cheng & Tsai, 2020; Georgiou et al., 2021; McCaw et al., 2021), including online environments (Duncan‐Vaidya & Stevenson, 2021). It is important to note that the novelty effect, the increased attention, and the effort associated with using new media (Clark, 1983), might have played a role in our students’ reporting of positive perceptions of immersive VR. Nonetheless, given that attitude and motivation are important in learning, using VR might be particularly beneficial for student with low self-efficacy for learning science (Cheng & Tsai, 2020) or interest in STEM, as one might expect for non-majors examined in this study. Our findings demonstrating overall positive perceptions of immersive VR in an *online course* are particularly timely given the increased use of online environments for learning in higher education and interest in VR as a teaching tool. Finally, given that attitudes towards technology are critical in their effective implementation and adoption into education (Halpin & Myers, 2002; Huang & Liaw, 2005), our work suggests immersive VR in online courses for non-majors is a promising opportunity for educators and education researchers.

### RQ2: VR Impact on Student Performance

Besides student perceptions, we also asked whether VR positively impacted student performance. Specifically, we examined whether assessment outcomes on the cardiovascular unit differed between semesters with and without a lesson in VR. We observed a small yet non-significant increase in student performance with the use of immersive VR lesson on the heart. We interpret this finding cautiously as the number of student participants in the study was small and we did not measure additional variables, such as prior knowledge, that may have influenced our students’ outcomes. However, recent work suggests there may be cognitive benefits to learning in VR (Di Natale et al., 2020; Makransky & Petersen, 2021) and that the positive perceptions of the technology we found can positively influence students’ conceptual learning gains (Georgiou et al., 2021). By providing students with a sense of presence or agency, VR can increase interest and motivation, all of which can enhance learning (Makransky & Petersen, 2021). The theory also suggests that the realism of visuals is important for teaching abstract concepts, and more realistic visual representations can improve student performance (Skulmowski et al., 2022). Indeed, generating a 3D mental image of an object from a 2D image, even with details and added realism of colors and shading, can be challenging for students (Skulmowski et al., 2022). With the sense of presence and ability to explore the 3D object, VR might enhance spatial knowledge of a given object and domain (Dalgarno & Lee, 2010; Maresky et al., 2019). Additionally, the construction of a 3D mental image from a 3D object might require less mental capacity, although the effect may depend on the students’ spatial ability (Huk, 2006). Because the human heart is anatomically complex, using a structurally realistic model in VR that can be observed from various angles and better represents the anatomy compared to a simple line drawing likely enhances learning. The use of immersive VR to learn about architecture, where students explored the exterior and interior of a virtual building, showed significant gains in architectural knowledge (Chan et al., 2022), suggesting the ability to see realistic objects in 3D aids student learning. Given the difficulty students have with the cardiovascular system all the way through medical school (Ahopelto et al., 2011; Kaufman et al., 2013; Södervik et al., 2019), experimental work is needed to better understand the benefits of different media, such as simple drawings, animations, and realistic 3D visuals on desktop and VR, in anatomy education.

### Instructors’ Notes on Benefits and Challenges

Finally, we documented instructor-perceived benefits and challenges. As recognized by others (Fromm et al., 2021; Nakai et al., 2022), we found that VR can afford various opportunities, such as “field trips,” in experiential learning. Furthermore, in agreement with previous work examining VR in online learning (Jeong et al., 2022), we found that immersive VR mimicked in-person interactions well, which was particularly noticeable for small group discussions. Instructors were able to move around the “room” and check in with different groups, as occurs in an in-person classroom setting. As Nakai et al. (2022), we found that VR benefitted our online course compared to Zoom alone. VR sessions allowed us to interact with all students in a more authentic way. We suspect that these social interactions in VR as well as the novelty of the technology influenced the overall positive perception students reported (see below). Future work should further examine how social interactions in VR contribute to the perceptions of the overall experience.

The benefits of teaching an online course in VR were also met with challenges and limitations, including nausea and internet issues, as has been noted by other studies (McCaw et al., 2021; Nakai et al., 2022). When internet speeds were less than the required speed (about 25Mbit/s), students experienced problems logging in and participating in the sessions, all of which suggest scalability problems with using VR in large online classrooms (> 50 students). Also, the implementation of immersive VR in an online course can highlight digital divide issues for urban vs. rural students and socioeconomic challenges when students might not be able to purchase or access the internet speed required to support the technology optimally.

### Future Directions

A study of VR technology in undergraduate science instruction is gaining momentum. Currently, most VR studies examine the use of VR in face-to-face classrooms, and more work is needed to explore the feasibility of the technology in a remote setting given the unique challenges encountered in online courses. We suspect that for many students taking online classes, the embodiment and the ability to have social interactions with peers and the instructor in VR provide motivation to participate and learn (Krämer, 2017), which could be directly assessed in future work with existing surveys on motivation. In addition to motivation, gauging student attitudes with open-ended questions about the VR technology over the course of the semester would elucidate other potential impacts on engagement and how it might impact performance. Finally, a comparison of how VR might influence attitudes and motivation towards science learning for non-major and major STEM students is needed to better understand the impacts the technology might have on recruitment and retention.

### Study Limitations

This pilot study offers insights into the feasibility and benefits of using VR in an online course; however, there are several limitations to the study. One limitation is that the data were collected from a single course at one institution, which might not be representative of student perceptions at research-intensive institutions or community colleges. Furthermore, we used closed-ended survey and evaluation prompts which limited gauging the breadth of perceptions students might have had about the VR experiences. Also, the students in the course were not science majors, which might influence their interest in the material presented in VR activities and thereby their ratings of the VR experiences. Lastly, given the continual advancement of VR, the challenges we identified might only capture the current state of the VR technology in online classrooms.

### Recommendations for Instructors

Ensuring internet speed is reliable and sufficiently high (greater than 25Mbit/s) is the first necessary step prior to considering the implementation of immersive VR activities in an online course. The choice of the hosting platform and content provider (or developer) should be weighed based on features available, customizability of avatars, environments, and objects as well as ease of use for an online course. Dedicating time to building up VR stamina to avoid dizziness is crucial, especially for those with no or minimal prior immersive VR experience. The time in the headset and in the VR environment, whether independent or as part of the class, is necessary to become physically and mentally more comfortable with the technology. If immersive VR is going to be a considerable part of an online course, we recommend encouraging the students to play games, watch 360° YouTube videos, or engage in “free-play” within VR to help students assimilate to the headset and controls prior to course activities. We also recommend the development of an Understanding and Agreement form for students to sign with a link to VR Oculus Health and Safety Warnings, to encourage students’ safe use of the technology indoors only and return of the equipment upon course completion.

## Conclusions

This study provides practical insights into the application of immersive VR activities (via head-mounted display) in an *online* course in higher education. Our work, in agreement with others, suggests VR has potential benefits for enhancing student learning and training, yet the research in the area is still new, and getting started poses many unknowns for educators. We encourage educators examining VR to share similar brief reports and provide conference presentations about their experiences with VR, platforms, and equipment used, in order for others to better gauge the benefits and feasibility of this tool. Time commitment and challenges associated with using immersive VR in an online classroom can appear discouraging, yet given the added benefit of social interactions and report of overall positive experience by students, educators should consider and weigh all of the costs and benefits.

## Supplementary Information

Below is the link to the electronic supplementary material.

Supplementary file1 (DOCX 85 KB)

## Data Availability

Data associated with the results are available on Figshare (10.6084/m9.figshare.22562335).
