# Supplementary material for: Using Immersive Virtual Reality in an Online Biology Course
Source: J STEM Educ Res. 2023 May 12:1–16. Online ahead of print. doi: 10.1007/s41979-023-00095-9 (PMC10175900; doi:10.1007/s41979-023-00095-9)
Supplement: Supplementary file 1 — Supplementary file1 (DOCX 85 KB) [file 41979_2023_95_MOESM1_ESM.docx]

**Supplementary Information**

To accompany *Using immersive virtual reality in an online biology course*

Journal for STEM Education Research

by Ania A. Majewska^1^ and Ethell Vereen^2^

^1^ Assistant Professor, Department of Physiology and Pharmacology, University of Georgia, Athens, GA 30602, USA

^2^ Assistant Professor, Department of Biology, Morehouse College, Atlanta, GA 30314, USA

Corresponding author: Ania A. Majewska, email: majewska@uga.edu

**I. Example lesson plans for sessions with virtual reality components**

**A. Cardiovascular unit**

Student learning outcomes (SLOs):

1. Describe the basic anatomy and the function of the heart
2. Describe how blood flows through the heart

| **SLOs/Time Allotted** | **Activity/Procedures** | **Materials/Notes** | **Assessment** |
| --- | --- | --- | --- |
| SLO1 & 2  Time: 30 minutes | Activity on Zoom  Students present for the online meeting are randomly assigned a part of the heart.  We use google draw and give students 5 minutes to familiarize themselves with the heart part they were given. Each student then labels the part of the heart they were assigned and briefly explain its function.  Heart parts:  superior vena cava  inferior vena cava  right atrium  tricuspid valve  right ventricle  pulmonary artery  pulmonary valve  pulmonary vein  left atrium  mitral valve  left ventricle  aortic valve  aorta  deoxygenated blood – color labels blue and move blue arrows  oxygenated blood – color labels bright red and move red arrows | PPT  And [google draw](https://docs.google.com/drawings/d/1VZp65yARsL81XTN6VXaqwTiJQVNeW-KXbQWo7LZnjjM/edit?usp=sharing) | Individuals label their assigned part of the heart and report to the class the function |
| SLO1 & 2  10 minutes | Activity in VR  Class meets on the virtual Morehouse campus quad where a virtual heart is present. Instructor highlights part of the heart visible from the outside and invites students to walk into the heart to examine the heart chambers. |  |  |
| SLO2  5 minutes | Video in VR  Short video on the flow of blood through the heart shown within the VR environment. |  | Quiz at end of VR session |

**B. Healthy Lifestyles unit**

Student learning outcomes (SLOs):

1. Investigate why certain foods are considered unhealthy
2. Examine 5 ‘healthy diets’
3. Weigh the benefits and drawbacks of the diets considered by the class
4. Reflect on challenges of healthy eating

| **SLOs/Time Allotted** | **Activity/Procedures** | **Materials/Notes** | **Assessment** |
| --- | --- | --- | --- |
| SLO1  Time: 10 minutes | Activity in Zoom  Students are asked to investigate why certain foods are considered healthy (e.g. whole grains) and others unhealthy (e.g. red meat). Also they answer additional question to prompt them to consider the difference between saturated and trans fats.  Students are provided websites (Mayo Clinic, webMD) on which to search for this information. | [Google docs](https://docs.google.com/document/d/1I3lTswaqFobgckrHJ4fGWZNnn-1r8WVJXTDNB479VlU/edit?usp=sharing) (Part 1)  Divide students randomly into 5 breakout rooms | Groups report back to class |
| SLO2  Time: ~30 minutes | Activity in Zoom (this can also be done outside class time)  Students investigate one of 4 “healthy diets” or popular eating trends:  1. Paleo diet  2. Keto diet  3. Vegan  4. Mediterranean diet  5. Military diet  Students are provided websites (Mayo Clinic, webMD) on which to search for this information.  Each group answers 7 questions about their diet that they present to the rest of the class later in VR. | [Google docs](https://docs.google.com/document/d/1I3lTswaqFobgckrHJ4fGWZNnn-1r8WVJXTDNB479VlU/edit?usp=sharing) (Part 2) | Groups report back to class. Student record responses on google docs. |
| SLO3  Time: 30 minutes | Activity in VR  Invite the students to a kitchen in which numerous food items are available. Instruct students to pick up food items that would be consumed as part of the diet they investigated and to bring the items to a dining room table labeled with that diet name. Note: in our VR environment the dining room was adjacent to the kitchen and students could easily move back and forth  Next, in the dining room, ask each group to pick a representative and explain what food items are on their table, and provide an overview of the diet. Ask the students to include answers to the questions from google doc (displayed on the wall of the virtual dining room).  Finally, ask students to ‘vote’ on what they consider the healthiest diet by moving their virtual body to the table labeled with the diet of their choice. | VR environment in an industrial kitchen and a dining room. Food items that pertain to the different diets (see SLO2).  Link to the google docs filled out by students as part of SLO2 | Responses to questions on google doc for each group and student explanation of the diets. |
| SLO4  Outside classroom | Activity outside the class  Students are asked write a reflection on the challenges they might be faced with, or have faced in the past) to eating healthy? |  | Individual reflections submitted on the learning management system. |

**II. Student feedback as provided by Qualtrics survey responses.**
